# T68. DIFFERENT INFLUENCES OF INTELLECTUAL FUNCTIONING AND COGNITIVE PERFORMANCE TO FUNCTIONAL OUTCOMES IN SCHIZOPHRENIA AND HEALTHY CONTROLS

**DOI:** 10.1093/schbul/sby016.344

**Published:** 2018-04-01

**Authors:** Leticia Czepielewski, Clarissa Gama, Cameron Carter, Angus MacDonald, James Gold, Steven Silverstein, Deanna Barch

**Affiliations:** 1 Universidade Federal do Rio Grande do Sul; 3 University of California, Davis; 4 University of Minnesota; 5 University of Maryland Baltimore; 6 Rutgers University; 7 Washington University

## Abstract

**Background:**

Individuals with schizophrenia (SZ) have marked functional impairments in wide range of domains, such as employment, independent living and interpersonal relationships. Several clinical, cognitive and psychological factors have been shown to predict functional outcomes. However, current pharmacological and psychosocial treatments have failed to rehabilitate patients, which indicates that the mechanisms of functional outcomes are not completely clear. Therefore, we aimed to better understand the relationship between intellectual and cognitive performance to functional outcome of subjects with SZ compared to unaffected individuals. Considering the neurodevelopmental course of SZ, our hypothesis was that premorbid crystallized IQ would interact with cognition to influence functionality.

**Methods:**

We included 188 individuals with confirmed diagnosis of SZ and 268 unaffected subjects (HC) from two separate multisite studies conducted by the Cognitive Neuroscience Test Reliability and Clinical Applications for Schizophrenia (CNTRACS) consortium. We used the following variables for the analysis: a) Estimated crystallized premorbid intellectual functioning (IQ): Wechsler Test of Adult Reading (WTAR); b) Cognitive performance: Dot Probe Expectancy task (DPX) and Relational and Item-Specific Encoding task (RISE) from CNTRACS; c) Functional capacity: UCSD Performance-based Skills Assessment – Brief (UPSA-B). We conducted linear regressions to predict functional outcome considering demographic, intellectual, and cognitive variables, as well as the interaction between cognition and IQ in participants with SZ and HC separately.

**Results:**

Participants with SZ had worse cognitive performance and premorbid IQ, and poorer functional outcome compared to HC. For the prediction of UPSA-B, the regression model that included cognition and IQ as predictors and age and parental SES as covariates was significant in SZ (F(4, 124) = 8.473, p < .001, Adj. R^2^ = .189), with the both variables showing significant main effects: IQ (β = .311, t = 3.324, p = .001) and cognition (β = .216, t = 2.630, p = .0010). When we included the IQ x cognition interaction (F(5, 123) = 7.035, p < .001, Adjusted R^2^ = .191), it did not significantly improve the model (F = 1.224, p = 0.27), and the interaction was not significant (β = -.11, t = -1.106, p = .27). In HC, the regression model with only main effects was similar to what was seen in SZ (F(4,313) = 27.62, p < .001, Adj. R^2^ = .25), with main effects of IQ (β = .239, t = 4.418, p < .001) and cognition (β = .349, t = 6.891, p < .001) (Figure 1). However, when we included the IQ x cognition interaction (F(5,312) = 24.15, p < .001, Adjusted R^2^ = .27), the interaction was significant (β = -.139, t = -2.801, p = .005) and accounted for a significant increase in variance over and above the other main effects (F = 7.8452, p = .005).

**Discussion:**

In SZ, both higher IQ and better cognitive performance were independent predictors of better functioning. However, in HC, functionality was predicted by the interaction between IQ and cognition, with the form of the interaction suggesting that for HC participants with higher IQ, there was less effect of cognition on predicting better functioning. Conversely, in HC with lower premorbid IQ, better cognitive performance has a stronger effect in predicting better functioning. The fact that both IQ and cognition had independent relationships to functional outcome in SZ could help explain the limited clinically significant results found in previous studies of cognitive remediation and psychosocial interventions that did not also consider the impact of premorbid IQ. Future studies could focus on early interventions to prevent functional impairments through the stimulation of early intellectual development.

